# Transition from three-port vats pulmonary resections to uniportal vats pulmonary resections: a single center experience

**DOI:** 10.1186/1749-8090-8-S1-O241

**Published:** 2013-09-11

**Authors:** JH Chung, YS Choi

**Affiliations:** 1 Department of Thoracic Surgery, Samsung Medical Center, Seoul, Korea

## Background

Although standard VATS approach is mostly performed through three or four incisions, recently uniportal VATS pulmonary resections have been reported to be a feasible and promising procedure. We report our initial experience with uniportal VATS.

## Methods

Data were retrospectively collected on patients who underwent uniportal VATS pulmonary resections in Samsung Medical Center between April 2013 and June 2013. Age, sex, diagnosis, anatomic resection, surgical technique and duration, incision size, complications, chest tube duration, length of hospital stay, reoperation, and mortality rate were examined.

## Results

10 major pulmonary resections (9 lobectomies and 1 segmentectomy) and 28 wedge resections (7 pneumothorax, 16 lung cancers, 2 interstitial lung disease, 1 hamartoma, 1 tuberculoma, 1 fibrous tumor of pleura) were performed by a single surgeon. With wedge resections, mean postoperative hospital length of stay, chest tube duration, and operative duration were 3 days, 2.07 days, and 69 minutes. With major pulmonary resections, mean postoperative hospital length of stay, chest tube duration, and operative duration were 6 days, 4.2 days, and 169 minutes.

There was one conversion to 3-port VATS during wedge resection due to thick pleural adhesion and one conversion to minithoracotomy during right upper lobectomy due to bronchial injury from technical error. There was no postoperative mortality or reoperation. One postoperative air leakage lasting longer than one week was noted after uniportal VATS right upper lobectomy which was resolved without any additional treatment.

## Conclusions

Although strict indications and some surgical tips might be needed in the initial period, uniportal VATS appears to be a safe and feasible procedure, which could be easily incorporated in diverse fields of thoracoscopic surgery without a long learning curve in established thoracic surgeons.

